# Publicity governance in contingency management during the COVID-19 pandemic in China: A “Government-Society” perspective

**DOI:** 10.1371/journal.pone.0293210

**Published:** 2023-11-10

**Authors:** Mengdi Wang, Xiaobing Peng

**Affiliations:** School of Public Policy and Administration, Chongqing University, Chongqing, China; East China Normal University, CHINA

## Abstract

**Background:**

Publicity is a common form of governance in government and is even more important in contingency management, especially during the COVID-19 pandemic. Publicity has two sides, the main body of publicity is led by the government, the object of publicity is the response to the public. So, publicity exists in the "government-society" field. Our aim was to find out how governments have been able to achieve effective publicity in the COVID-19 and to promote the active participation of society in governance.

**Materials and methods:**

We adopted a case study approach. Taking Chongqing Municipality as an example, we collected 201 messages from five forms of publicity, including cell phone SMS, village (community) broadcast, publicity placards or banners, official websites, and government media platforms during the period of 2020–2021, and described and analyzed the publicity content of different forms of publicity.

**Results:**

During the whole period of the COVID-19, the publicity governance under contingency management showed the characteristics of focusing on administrative efficiency, social efficiency and post-social efficiency, and showed specific publicity governance functions such as mobilization and control, education and clarification, cultivation of public consciousness and shaping government credibility.

**Conclusions:**

Publicity governance in contingency management during the COVID-19 pandemic emphasizes three effective approaches: time synchronization, organizational scale, and interaction among multiple advocacy agents. This can extend the existing government-centered research to the level of interaction between the government and society, and help the government to better use effective publicity to achieve the governance task under contingency management.

## 1. Introduction

In normalized government governance, publicity, as a social control method that manipulates mass behavior through carefully calculated information dissemination, has gradually become a new channel for the government to convey its will and publicize the purpose of social governance [1], with emphasis on the control role of publicity [2]. For example, after the war, the government changed its publicity strategy to change international relations [3]; the government satisfies the requirements of the higher government through publicity on the Internet which serves as a tool for the government to strengthen control [4], so that the publicity structure and role established by the government can be regarded as a thought control system. However, with the update of new media technology, publicity also plays a role of in citizens’ participation in government governance in modern government governance, to form public trust in government and promote the transformation and promotion of government [5].

Publicity is especially prominent in the prevention and control of COVID-19. On the one hand, during the COVID-19 pandemic, the government’s publicity is helpful to publicize prevention measures and prevention rules, and promote the government’s control and prevention of the COVID-19 pandemic, and gain other political benefits by doing so [6, 7]. Such as effectively responding to the public’s emotional demands and increasing the public’s trust in the government [8–10], accelerating the widespread public vaccination of the COVID-19 vaccine [11], and even gain favorable election resources through publicity [12, 13]. On the other hand, the pandemic will prompt people to pay close attention to news and information. Due to the disorder of information, the public will receive a large amount of information that cannot be judged as true or false, leading to bad psychological emotions and social reactions [14–16], therefore, the positive and authoritative publicity of the government can effectively explain and contain false news and rumors [17–19]. In addition, the media tools used in government publicity also promote the prevention and control of COVID-19 by the government and the public. For example, the advanced Internet public health publicity model enables the public to grasp the basic information and prevention methods of COVID-19 [20]. The wide application of new mobile media devices enables the public to easily access information propagated by the government and express their views and emotions [21, 22], Weibo (“Facebook in China”) also served as a tool and mainstream media for the government to publicize public policies on COVID-19 prevention and control [23].

However, we need to pay more attention to the collaboration between the government and society in the publicity process. Specifically, the publicity adopted by the government is related to its authority, which can be regarded as a kind of meritocracy [24, 25], but this view focuses on the government field and ignores some realistic situations, such as: The publicity tool of the government is the media platform facing the social public, which exists in the society and promotes the government’s governance of the COVID-19 pandemic [21]. As a way of communication between the government and society, publicity, as a means of communication between the government and society, feedback on the government’s public service supply and public governance [26], requires attention to the social significance of the government’s publicity and governance under contingency management [27].

Therefore, the purpose of this study is to find out how the government achieved effective publicity during the COVID-19 pandemic through the case analysis of Chongqing in China, and to promote the active participation of the society in government governance.

## 2. Theoretical perspective: Publicity governance in government and society

Contingency management reveals the unconventional characteristics of publicity governance, and these characteristics also reflect and act on the process and action of publicity governance of sudden major public events. The governance system consists of two parts: the government governance system and the social governance system [28]: the government governance system relies on laws, regulations and administrative levels to regulate the responsibilities of governments at all levels, while the social governance relies on government mobilization to adjust public behaviors [29]. This constitutes two activities and fields of action of publicity governance during the COVID-19 pandemic: government and society. In different fields, publicity has different roles and functions.

On the one hand, the government field emphasizes the publicity and governance of the modern administrative level and traditional discipline. The publicity governance in the government field depends on the bureaucratic structure of administrative organizations, which can convey publicity information at the fastest rate and to the maximum extent [30]. In the bureaucratic structure chain of administrative organizations, there are two publicity governance modes: modern administrative level and traditional discipline [31]. However, these two forms of publicity governance are both within the administrative framework of the government field. The unconventional characteristics of contingency management require different levels of government to maintain the efficient consistency of information and publicity, so that the village (community) can adapt to the overall unity and coordination of publicity governance by amplifying traditional disciplining methods. It is a governance mode that emphasizes “motivation” [32].

On the other hand, publicity in the social field is oriented towards the interests of the public, which is a tool for realizing the value of government governance. Publicity is not only a kind of information practice, but also an important carrier of social communication. The status quo of government governance can be understood from the content or form of publicity. publicity governance in the social field is oriented to the interaction between the government and society, which is realized through the dual value of governance [31]. In the context of COVID-19 pandemic, publicity governance in contingency management is more of an explicit instrumental and invisible publicity, as well as an unconventional feature of achieving publicity through instrumentality. That is, publicity governance, as a tool technology or means under social field governance, is externally manifested as requirements and mandatory requirements for the public. Its intrinsic nature is the maintenance of public interests in the context of major public emergencies [33, 34]. This is a publicity governance mode limited by a crisis situation and risk aversion. Under this condition, publicity governance in the social field pay more attention to publicity of pandemic and response to social requirements.

## 3. Materials and methods

**Fig 1:**
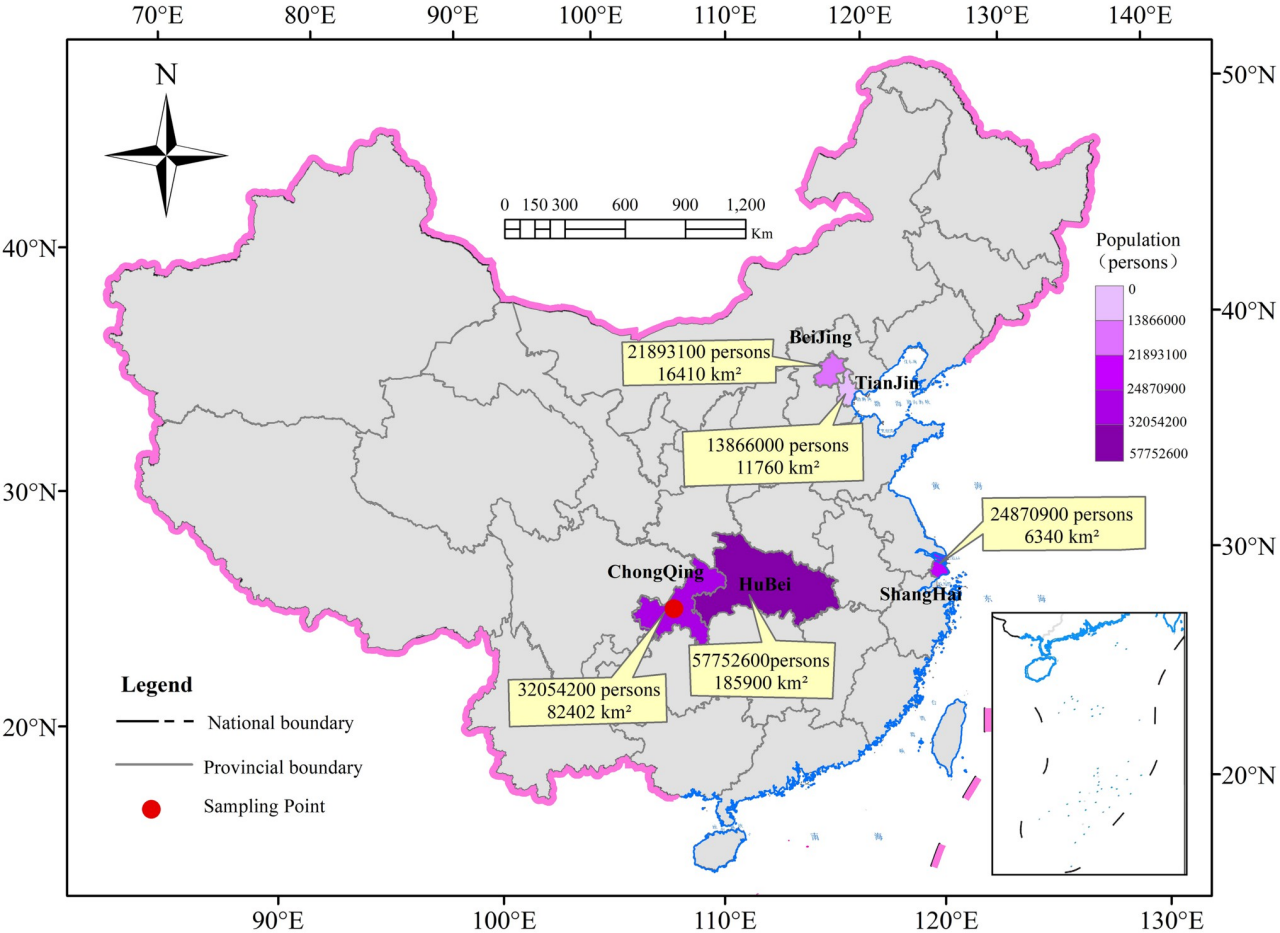
Map of the location and situation of four municipalities in China and Hubei Province in which the pandemic initially occurred. Source of original data on resident population and area size of regions: Bulletin of the 7th National Population Census (NPC) of China; https://www.gov.cn/guoqing/2021-05/13/content_5606149.htm, and Statistical Tables of Administrative Divisions of the People’s Republic of China; https://xzqh.mca.gov.cn/statistics/2022.html.

This study adopts the case study method, supplemented by descriptive analysis. Chongqing is selected as the research object for the following reasons: (1) Chongqing (one of the four municipalities directly under the Central Government in China) is the most populous city in China, with a population of more than 32 million in 2021, significantly larger than Beijing and Wuhan, the earliest outbreak city in China, and is the largest municipality in terms of area, equal to 2.4 times the area of the other three municipalities combined. In addition, Chongqing is adjacent to Hubei Province, where the outbreak occurred, and 53 townships in nine districts and counties in Chongqing share a border with Hubei Province (Fig 1). It has close demographic and economic interaction with Hubei Province. Municipalities are characterized by a large resident population, a high level of urbanization, and a high concentration of industries and population. Municipalities are important in China’s political, economic, social, cultural, scientific and technological innovation, and transportation sectors, and their development trends, policy directions, and governance experience often influence all aspects of China; there are currently four municipalities in China, namely, Beijing, Tianjin, Shanghai, and Chongqing. At the beginning of the COVID-19 pandemic, Chongqing was rapidly spreading and soon became one of the top ten hardest hit areas in China due to its geographic location, urban culture, economy and trade, and population. Obviously, Chongqing faced a huge risk of epidemic spread and its publicity and management pressure was even more prominent; (2) Chongqing is one of only four municipalities directly under the central government in China, which can directly accept the management of the central government, and can also directly implement the central policy to the local and grassroots level, having one less administrative level than other provinces, which can enable us to collect and analyze the information in a more comprehensive way; (3) during the pandemic period, all the provinces and municipalities carried out the relevant publicity in accordance with the policy of the central government and the development of the epidemic, and the overall work had a greater degree of homogeneity. And choosing Chongqing as a research sample enables us to collect information better. Therefore, we chose Chongqing as a single city as the object of the study.

We selected Chongqing and used the method of case study and description analysis to collect data from 2020 to 2021 (when the COVID-19 pandemic had been controlled and entered normal management in Chongqing at the end of 2021). The publicity measures and contents carried out by the Chongqing government during the prevention and control of the COVID-19 pandemic mainly include two parts: publicity methods and publicity contents. In terms of publicity methods, the publicity methods used were analyzed according to relevant publicity literature [13, 19], and the actual situation of many villages (communities) in China, we divide into five categories: mobile phone short messages, village (community) broadcast, publicity placards or banners, official website, Weibo or official account and other government media platforms (such as Chongqing CDC, Chongqing government information release, etc.). The publicity content mainly focuses on what is expressed in different publicity methods, such as COVID-19 prevention and control measures, the infection spread data, government measures, correction of rumors or false news, etc. Of course, due to the huge and diverse spread of COVID-19 information, we cannot collect all the information comprehensively, and we focus on the content of the publicity and its inherent significance. Therefore, when collecting information, we also excluded similar information with very similar publicity content and publicity methods as well as routine COVID-19 data publication (Chongqing Municipal government will publish COVID-19 pandemic data every day), which will not affect our research.

On this basis, we collected a total of 201 pieces of information under five publicity methods, including mobile phone short messages, village (community) broadcast, publicity placards or banners, official website, government media platforms, and described and classified the publicity content under different publicity forms. Then, from the perspective of the "government-society" field, we will study the key contents and characteristics of Chongqing government publicity and governance at different stages of COVID-19 prevention and control, taking into account the publicity situation in Chongqing during the COVID-19 pandemic. Finally, we will comprehensively analyze the logic of the government’s publicity and governance under contingency management during the COVID-19 pandemic.

## 4. Results

**Fig 2:**
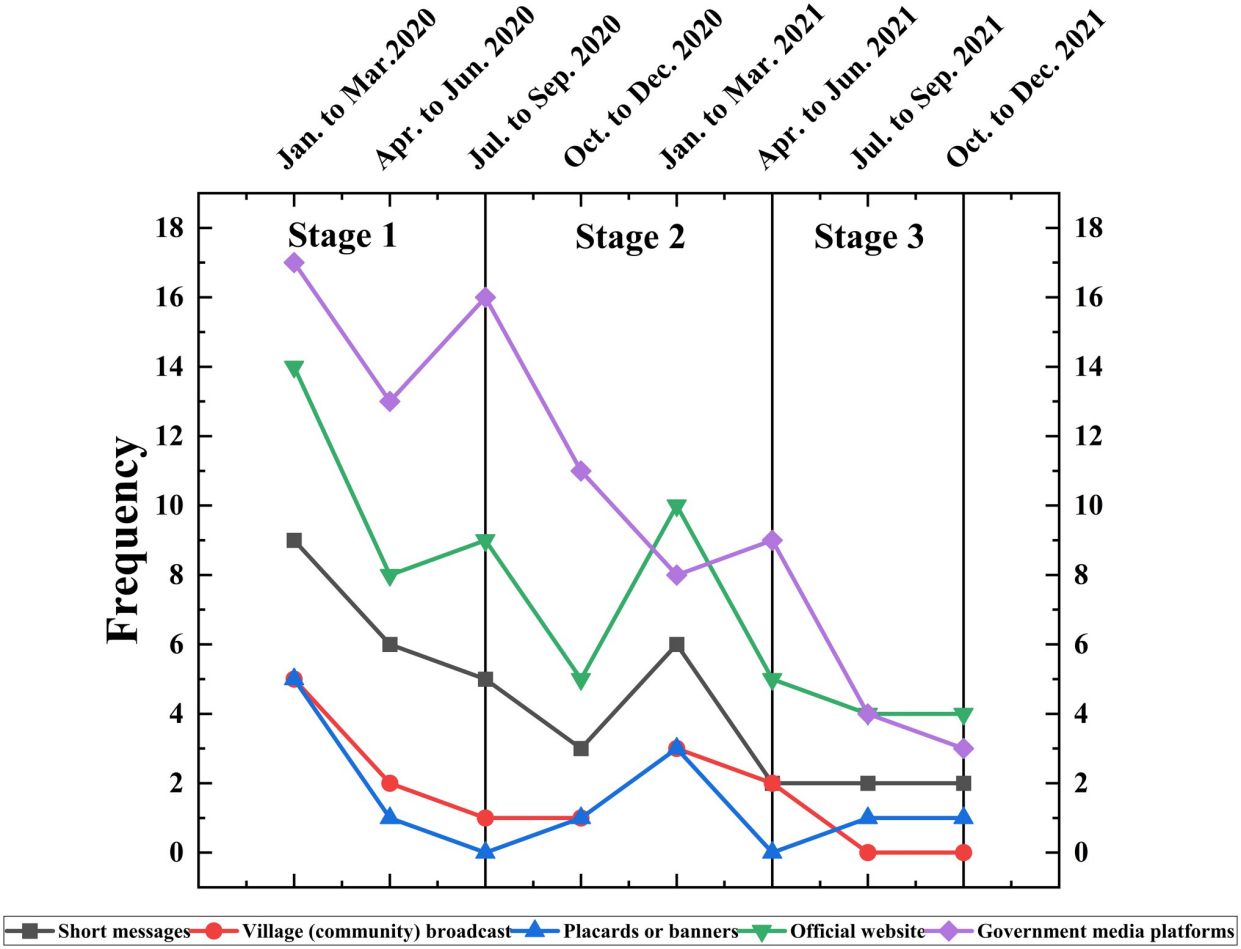
Trend of different publicity ways during the COVID-19 pandemic.

According to the five different methods of publicity and divided by quarterly time, we made a statistical analysis of the publicity content of Chongqing Municipal Government during the COVID-19 pandemic in China (Table 1). Statistical analyses here mainly utilized data obtained from daily detection records or specialized surveys, grouped by different times or different types, to describe relevant characteristics or trends. By collecting the mode and content of publicity in Chongqing Municipality during the COVID-19 period, we conducted descriptive analyses in terms of both historical review and structural profiling. The results showed that the publicity on official websites and government media platforms accounted for a relatively large proportion, accounting for 59.4% of the total publicity content. At the same time, the number of government Publicizing of Chongqing Municipal Government showed a decreasing trend in terms of time, which is consistent with the progress of COVID-19 prevention and control work. It can be roughly categorized into three phases (Fig 2): from January to September 2020, from October 2020 to June 2021, and after July 2021. It is worth noting that the data for January-March 2021 picked up, mainly due to the arrival of the Chinese New Year, which boosted the Chongqing Municipal Government’s publicity on COVID-19 prevention and control efforts.

**Table 1:** Statistical results of different publicity ways during the COVID-19 pandemic.

Time	Short messages	Village (community) broadcast	Placards or banners	Official website	Government media platforms	Total
Jan. to Mar.2020	9	5	5	14	17	50
Apr. to Jun. 2020	6	2	1	8	13	30
Jul. to Sep. 2020	5	1	0	9	16	31
Oct. to Dec. 2020	3	1	1	5	11	21
Jan. to Mar. 2021	6	3	3	10	8	30
Apr. to Jun. 2021	2	2	0	5	9	18
Jul. to Sep. 2021	2	0	1	4	4	11
Oct. to Dec. 2021	2	0	1	4	3	10
Total	35	14	12	59	81	201

### 4.1 Stage 1: Emphasis on administrative efficiency

At this stage, when the COVID-19 pandemic is at its peak, the number of publicity methods such as mobile phone short messages, village (community) broadcast, publicity placards or banners, official website, and government media platforms accounted for 57%, 57%, 50%, 52.5% and 56.7% of the total publicity methods. In, through information publicity and communication within the organizational structure, Chongqing has implemented hierarchical and precise joint prevention and control measures in a short time, which has played a key role in pandemic prevention and control, reflecting the efficiency of the modern administrative level [35–38]. At the same time, at the end of the administrative organization structure, that is, the village (community) level, the publicity governance of COVID-19 pandemic prevention and control adheres to the spirit and requirements of the higher government, but in the form of publicity governance, it reflects the characteristics of a compliant traditional discipline, and an irrational and even overcorrected publicity form, that is, publicity and mobilization and control.

In contingency management, the publicity management of the government acts above all means to mobilize masses of broad people. Mobilization focuses on the meaning of behavior choice (possible or impossible) for others or mobilization objects, and uses coercive orders and motives to persuade the construction of the motivation of mobilization objects, to achieve social control by conforming to the requirements of mobilization [39]. In the whole process, publicity and management communicated information with high efficiency and strictness under contingency management, and mobilized the masses in various forms to carry out home isolation and self-prevention following the prevention and control requirements. The extensive, important, imperative and persuasive nature of publicity and management information could fundamentally mobilize mass actions to control the spread of the pandemic. The establishment of a national “defense line” for the outbreak of COVID-19 has achieved the first time and first action for pandemic prevention and control.

For example, the “hardcore publicity of the village head” (strongly urging residents to stay in isolation and not to go out), “closing the road and closing the village to copy the homework” (learning the COVID-19 prevention and control measures in other regions), some colloquial banners that combine jokes and demands (e.g., visiting is harmful, gathering is suicide), and text messages on how to stay at home. In this form of publicity, people are strictly restricted or required to behave.

### 4.2 Stage 2: Focus on social efficiency

At this stage, the COVID-19 pandemic in Chongqing was gradually under control, and the publicity of relevant information was gradually reduced. Emphasis will be placed on public preparedness during Chinese holidays, such as National Day and Spring Festival. At this stage, the main task of Chongqing government’s publicity governance turns to the education and guidance of public opinion to maintain social stability. Specifically, there are two aspects:

#### Education and regulation in the field of government

With the update and development of information technology, new media make publicity present the characteristics of fragmentation, selective interpretation, the invisible influence of communication form and amplification effect of communication effect [40]. Especially in the publicity and reporting of risk crisis events, public panic will increase at the initial stage of the event [41, 42]. Therefore, in the context of the development of new media and information asymmetry, the two main tasks of publicity governance in contingency management are to educate the public about the COVID-19 pandemic and standardize the dissemination of undesirable information (mostly rumors) [43]. In the process of publicity governance of the COVID-19 pandemic, the municipal government explained the COVID-19 pandemic, reported the progress of the COVID-19 pandemic, and invited authoritative figures to “dispel rumors” about the pandemic prevention and control work through TV, and the accuracy, scientific and universality of publicity information during the COVID-19 pandemic should be standardized.

#### Clarifying social doubts in the social field to maintain stability

In the social field of contingency management, the extension of publicity governance is more extensive, including the governance of the emotions and behaviors of the public, social media and all kinds of information in the progress of events [44]. In the process of COVID-19 prevention and control, social panic caused by “shutting down cities and villages”, rampant buying caused by rumors of “Shuang Huang Lian suppressing the virus” have brought great side effects to COVID-19 prevention and control and social stability. At this time, a comprehensive, authoritative and scientific pandemic event is presented to the public through a multi-channel publicity method, including experts refuting rumors, pandemic interpretation, constant cure news and virus research progress information. The relevant facts are clarified, the public emotions are calmed, the progress of the COVID-19 pandemic is popularized, and the social order is stabilized. It has provided a rational and orderly social governance environment for the prevention and treatment of the COVID-19.

### 4.3 Stage 3: Advocating “post-social” efficiency

At this stage, the COVID-19 pandemic has entered the period of normal management. The focus of Chongqing government’s publicity governance is on how to form a good social response ability and mechanism through the prevention and control of the COVID-19 pandemic.

#### Cultivate public awareness through publicity

There is an internal consistency between governance innovation and public consciousness [45]. Especially under the background of contingency management, the requirement of compulsory action to the public is the basic norm in contingency management. The higher the conscious awareness of the people, the weaker the coercive publicity is. It can even be regarded as the coercive management of publicity is the cultivation of the conscious awareness of the people in contingency management. In the prevention and control of the COVID-19, information campaigns related to the public, such as “staying at home is participating in pandemic prevention”, “The Most Beautiful Traveler”, “Go Chongqing, Go China”, were used to appeal and advocate the public’s social responsibility and rational resonance for participating in the prevention and control of the COVID-19 [46, 47]. And then cultivate the rational and conscious consciousness of the public in contingency management.

#### Shape the credibility of the government to achieve social resonance

In contingency management, when the local government is late or inefficient, it is easy to affect the social public’s resistance [48]. At this time, the main task of publicity governance in the social field is to shape the credibility of the local government [45]. In the publicity governance of the COVID-19 pandemic, the government’s official media have made positive responses to the prevention and control of the COVID-19 pandemic, including the publicity of excellent prevention and control areas, the report of national medical support, and the publicity of information such as government press conferences or interviews with major local leaders, which has shaped the credibility of local governments. Furthermore, the shaping of credibility is based on the response to and satisfaction of social expectations, which is consistent with the psychological expectations of the public, and is easy to form a resonance effect between society and local governments in the prevention and control of the COVID-19 [49–51]. Publicity governance enables the public to gain a sense of belonging, sense of responsibility and security from the local government’s COVID-19 prevention and control actions, comply with the government’s guidance, consciously participate in the COVID-19 prevention and control work, and generate collaborative forces [52, 53].

## 5. Conclusions

**Fig 3:**
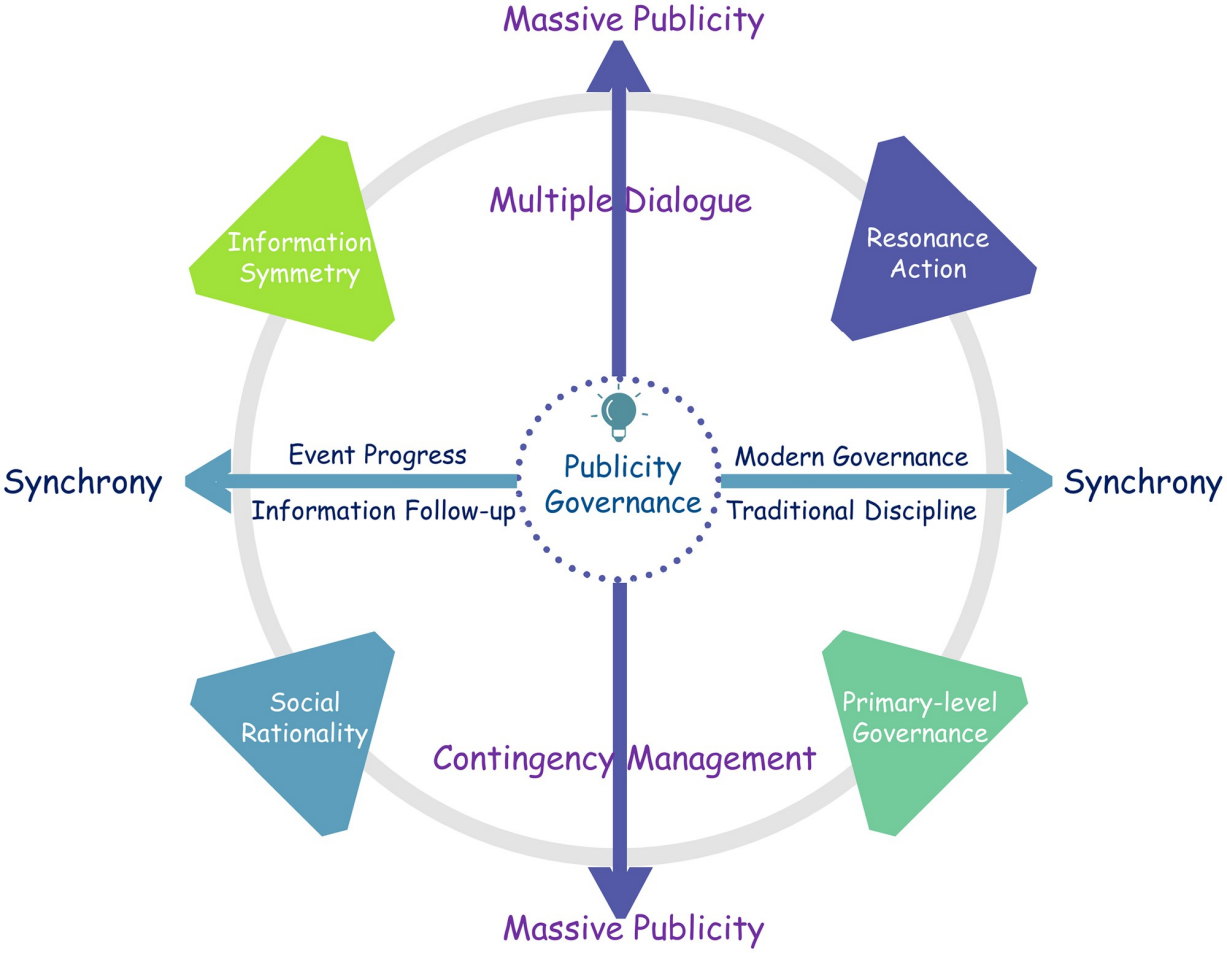
The logic model of publicity governance in contingency management during the COVID-19 pandemic.

In contingency management, publicity governance plays a role in the government field and the social field. The operation logic behind such publicity governance is reflected in a horizontal and vertical logical structure, that is, the synchrony logic in the horizontal time dimension, the massive publicity logic in the vertical dimension, and the interactive logic between the two (Fig 3).

### 5.1 The logic of transverse time dimension: Synchrony

On the one hand, the synchrony of event progress and information follow-up. The production of COVID-19 information mainly involves four subjects: local government, the public, event reality and social media. Different information production rates among subjects will lead to dilemmas such as “information vacuum” or “information misinformation” [15, 54], causing obstacles to the prevention and control of COVID-19. Therefore, this synchronicity is the need of society in the social field, the requirement for the public and the prevention and control of COVID-19 in the case of asymmetric information, and the reflection of the role of publicity governance in social thinking and social cognition.

On the other hand, the synchrony of modern governance and traditional discipline. In the field of publicity governance rely on an administrative organization structure that realizes the top-down information or action command, but in the grassroots community, due to the traditional management of rural moral habits, the new crown outbreak management ability weak status quo, such as traditional discipline action has become an important way, community publicity governance their government hierarchy governance requirements on this way, action, such as maintain synchronic, In this way, we can construct a top-down and synchronous publicity governance action, which is a reflection of the overall governance of the government in the context of the COVID-19 pandemic.

### 5.2 The logic of vertical structural dimensions: Massive publicity

Massive publicity means that the government mobilizes all departments to do it together, forming a governance mode of interworking, horizontal joint and joint management, and emphasizing the dialogue and communication of multiple subjects participating in publicity. Especially in the urgent administrative background, the publicity governance can better reflect the governance ability of the country under the emergency major public events.

On the one hand, massive publicity promotes dialogue and communication among multiple subjects. The publicity governance of major public emergencies requires the participation of multiple departments. However, there are differences in management functions and operation modes among different departments, and how to make up for the publicity obstacles caused by such differences requires a publicity principle of dialogue and communication. This principle not only means that each department provides necessary resources and implementation measures for publicity governance, but also requires each department to assume and play its own publicity function.

On the other hand, massive publicity means an orderly communication order. The key of governance is to establish an orderly relationship among different interest groups. In publicity governance, this orderly relationship is the communication order. Massive publicity is an important way to realize this orderly communication order. In the governmental field, publicity governance is carried out according to legal rules, while in the social field, publicity governance is carried out with traditional rationality as the starting point. The combination of rationality and jurisprudence unifies publicity governance in the government and social fields.

## 6. Discussion

Synchrony and massive publicity constitute the horizontal and vertical structure of publicity governance under urgent governance, and there is an interaction between them. This means that the publicity subject and information object, event information and social information, government and society are incorporated into the role and implementation framework of publicity governance, reflecting the role of publicity governance in the COVID-19 prevention and control, to achieve effective publicity and governance under contingency management. There are three main implementation steps: The first is to realize the symmetry of events and social information. Multiple subjects can play the role of dialogue and communication in large-scale publicity, and synchronic publicity on the COVID-19 can make up for the problem of asymmetric social information. Second, realize social rationality. Multiple authorities and Official communication staff need to shoulder the responsibility of publicity governance under the COVID-19 pandemic, maintain public order and maintain social rationality. Third, achieve common action. The consensus between the government and society on coping with the COVID-19 pandemic is formed through publicity governance, and to promote coordinated and synchronic joint action between government and society.

Further, during the COVID-19 pandemic, this effective way of publicity under the contingency management was not only practiced in Chongqing, but also in other regions. In other Chinese cities, such as Beijing, Shanghai and Wuhan, local government departments also carry out publicity through official websites, social media, short messages and other channels. Meanwhile, in China’s huge rural areas, rural leaders carry out effective publicity through radio [55], and to help farmers participate in government. In less developed areas, modern media means have become the main means of publicity during the COVID-19 pandemic [7, 18, 20], however, in areas with weak information infrastructure, publicity forms such as short messages and radio can significantly improve the information perception effect of "individuals with limited Internet access" [56]. Therefore, during the pandemic in China, the logic of realizing effective publicity governance under emergency management can also be understood as an interactive logic: in the whole process, firstly, we need the government to make comprehensive use of modern and traditional publicity tools; secondly, the government should give corresponding rights and responsibilities to grass-roots villages and community organizations, and incorporate them into diversified actions; and lastly, in accordance with the principle of orderly dissemination, it should vigorously promote their social actions in coordination with the state’s requirements and governmental behaviors.
